# Failure Analysis of Acid-Thinned Coiled Tubing Under HTBH Conditions: Role of Inhibitor Depletion and Corrosion Asymmetry

**DOI:** 10.3390/ma19153200

**Published:** 2026-07-27

**Authors:** Marko Kršulja, Lovro Liverić, Damir Karabaić, Vedrana Špada

**Affiliations:** 1 Faculty of Engineering, Juraj Dobrila University of Pula, Negrijeva 6, 52100 Pula, Croatia; marko.krsulja@unipu.hr (M.K.); damir.karabaic@unipu.hr (D.K.); 2 Metris Research Center, Istrian University of Applied Sciences, Preradovićeva 9D, 52100 Pula, Croatia; vspada@iv.hr

**Keywords:** coiled tubing failure, HTBH acidizing, pitting corrosion, inhibitor depletion, corrosion asymmetry, hydrogen embrittlement, ductile fracture

## Abstract

**Highlights:**

**What are the main findings?**

- External wall loss was ~12 times greater than internal loss in the final operation.
- Spent-acid backflow caused uniform external corrosion; internal attack was pitting.
- The tubing failed by ductile overload of a corrosion-thinned section.

**What are the implications of the main findings?**

- HTBH inhibitor testing should reproduce spent-acid backflow conditions.
- Inner and outer tubing surfaces should be assessed separately.
- Wall thickness and pit depth should be checked before tubing reuse.

**Abstract:**

A CT-80 coiled tubing fractured at the gooseneck during retrieval after a 2.5 h treatment with 15% HCl under high-temperature bottom-hole conditions (196 °C). The failure was investigated by dimensional measurements, metallography, Vickers microhardness testing, SEM/EDS, and FT-IR spectroscopy. Pronounced corrosion asymmetry was observed. Cumulative external wall loss reached 1.196 mm, compared with 0.292 mm on the inner wall, while the wall loss attributed to the final operation was approximately twelve times greater externally than internally. These findings suggest two different exposure histories: predominantly uniform attack of the outer wall during backflow of spent, inhibitor-depleted acid, and localized pitting of the inner wall under incomplete inhibitor coverage. EDS mapping identified Sb-rich deposits around inner-wall pits. In combination with the relevant literature, this distribution is consistent with a possible Sb–Fe galvanic effect that may have promoted local anodic dissolution, although galvanic coupling was not measured directly. The FT-IR spectra were consistent with iron oxides/oxyhydroxides, carbonate-containing scale, sulfate-bearing products on the outer surface, and thin organic residues rather than a continuous inhibitor film. Microhardness increased from 229 HV1 in the new tubing to 243.4 HV1 at the fracture location; this increase may reflect limited hydrogen uptake together with service-induced strain hardening or residual stresses. Fractography showed necking and dimpled microvoid coalescence, supporting a predominantly ductile overload mechanism in the corrosion-thinned section. A limited contribution of hydrogen to ductility loss cannot be excluded because the hydrogen content was not quantified.

## 1. Introduction

Steel tubular components used in oil and gas production are frequently exposed to acidic environments generated by matrix acidizing treatments, co-produced H_2_S and/or CO_2_, and injected CO_2_ [1,2]. Among these conditions, hydrochloric-acid stimulation is particularly aggressive because carbon steel corrosion in strong acid is driven by anodic iron dissolution coupled with cathodic hydrogen evolution [3,4,5,6]. In coiled tubing operations, this exposure is not necessarily symmetric. During acid injection, the inner wall is contacted by relatively fresh inhibited acid, whereas during backflow the outer wall may be exposed to spent acid that has reacted with the formation, lost part of its inhibitor content, and remains highly corrosive at low pH [7,8,9]. This difference between the inner and outer surfaces is important because it can produce different corrosion morphologies and different rates of wall loss within the same component.

The severity of acid corrosion increases strongly with temperature, which makes high-temperature bottom-hole operations especially demanding [10]. At temperatures above about 100–120 °C, conventional inhibitor packages may lose efficiency, and acid treatments therefore require carefully selected organic inhibitors and intensifiers [11,12,13,14,15,16,17,18,19]. Propargyl alcohol and quaternary ammonium compounds are widely used because they can adsorb on steel and form protective organic layers, while antimony-based intensifiers are added to extend protection under more severe acidizing conditions [20,21,22,23,24,25,26,27,28,29,30,31,32,33,34,35]. However, inhibitor performance depends on concentration, acid strength, temperature, flow conditions, surface state, and exposure time. If the inhibitor film is incomplete or depleted, localized attack may start even when the bulk acid was initially inhibited.

The presence of H_2_S and CO_2_ further complicates the failure mechanism. H_2_S can increase corrosion severity and promote hydrogen entry into steel by suppressing recombination of atomic hydrogen into molecular hydrogen [36,37,38,39,40,41,42]. This may lead to hydrogen-induced cracking, sulfide stress cracking, or reduced ductility, especially when mechanical stresses are present. CO_2_, on the other hand, can contribute to carbonate corrosion products such as siderite-type scales. In acidizing operations, these chemical effects interact with coiled tubing mechanics. Coiled tubing is repeatedly bent and straightened during service and experiences combined tensile and bending loads while passing over the gooseneck. Therefore, even moderate corrosion damage can critically reduce the load-bearing cross-section and shift the final failure mode toward overload.

Failure analysis of coiled tubing must therefore distinguish between several possible mechanisms: uniform acid thinning, localized pitting, corrosion-assisted fatigue, hydrogen-assisted brittle fracture, and ductile overload. A reliable interpretation cannot be based on visual inspection alone. It requires dimensional measurements to quantify wall loss, metallography to assess microstructure and defects, hardness testing to evaluate possible hydrogen-related changes, SEM fractography to identify the fracture mode, and chemical analysis of corrosion products to reconstruct the exposure history.

Although corrosion inhibition of carbon steels in acidizing environments has been extensively studied, full-scale failure investigations that quantitatively distinguish between inner- and outer-wall damage in coiled tubing after HTBH acidizing operations remain limited. In particular, the combined effects of spent-acid backflow, inhibitor depletion, Sb-containing intensifiers, and mechanical loading during passage over the gooseneck have not been sufficiently resolved using integrated dimensional, chemical, metallographic, and fractographic evidence. Consequently, it is often difficult to distinguish hydrogen-assisted fracture from ductile overload of an asymmetrically corrosion-thinned section.

The present work addresses this gap through the investigation of CT-80 coiled tubing that fractured at the gooseneck during retrieval after a 15% HCl matrix-acidizing treatment performed at a bottom-hole temperature of 196 °C. Dimensional analysis, optical metallography, Vickers microhardness testing, SEM/EDS, and FT-IR spectroscopy were combined to reconstruct the exposure history and determine the dominant failure mechanism. Particular attention was given to the difference between inner- and outer-wall corrosion, the possible consequences of inhibitor depletion during backflow, the association between Sb-rich deposits and localized pitting, and the distinction between hydrogen-assisted damage and ductile overload of a corrosion-thinned section.

## 2. Materials and Methods

### 2.1. Operational Background and Field-Failure Conditions

The investigated material was commercially supplied CT-80 coiled tubing intended for well-intervention service. The tubing had a nominal outer diameter of 31.75 mm, a nominal wall thickness of 2.59 mm, and a specified minimum yield strength of 552 MPa. It had been used in field operations before the final acidizing treatment investigated in the present study. Pre-operation dimensional inspection indicated that wall loss was already present before the final operation, as quantified in Section 2.2. Metallographic examination identified a longitudinal manufacturing seam, which was used to orient the cross-sections and define the 12:00 reference position for the microstructural and microhardness examinations.

During the final acidizing operation, a total volume of 7 m^3^ of working acid containing 15% HCl was pumped through the coiled tubing. The working acid contained a commercial inhibitor package based on propargyl alcohol, quaternary ammonium salts, antimony trioxide, and formic acid. The commercial inhibitor package was mixed with the working acid before pumping through the coiled tubing. The treatment lasted 2.5 h, and the reported bottom-hole temperature at the treatment interval was approximately 196 °C.

The fracture occurred in service during retrieval of the coiled tubing, rather than during laboratory mechanical testing. It was located approximately 100 m from the lower end of the deployed tubing. For specimen designation in the present study, the fracture location was defined as 0 m, while the second service-exposed specimen was collected approximately 100 m above the fracture location. During passage over the gooseneck, the tubing was subjected to combined axial retrieval tension and bending. At the wellhead, 300 ppm H_2_S was detected using a Dräger instrument (Dräger Safety AG & Co. KGaA, Lübeck, Germany), while the CO_2_ content of the gas phase was 11.29%.

### 2.2. Sample Appearance and Dimensional Analysis

**Figure 1:**
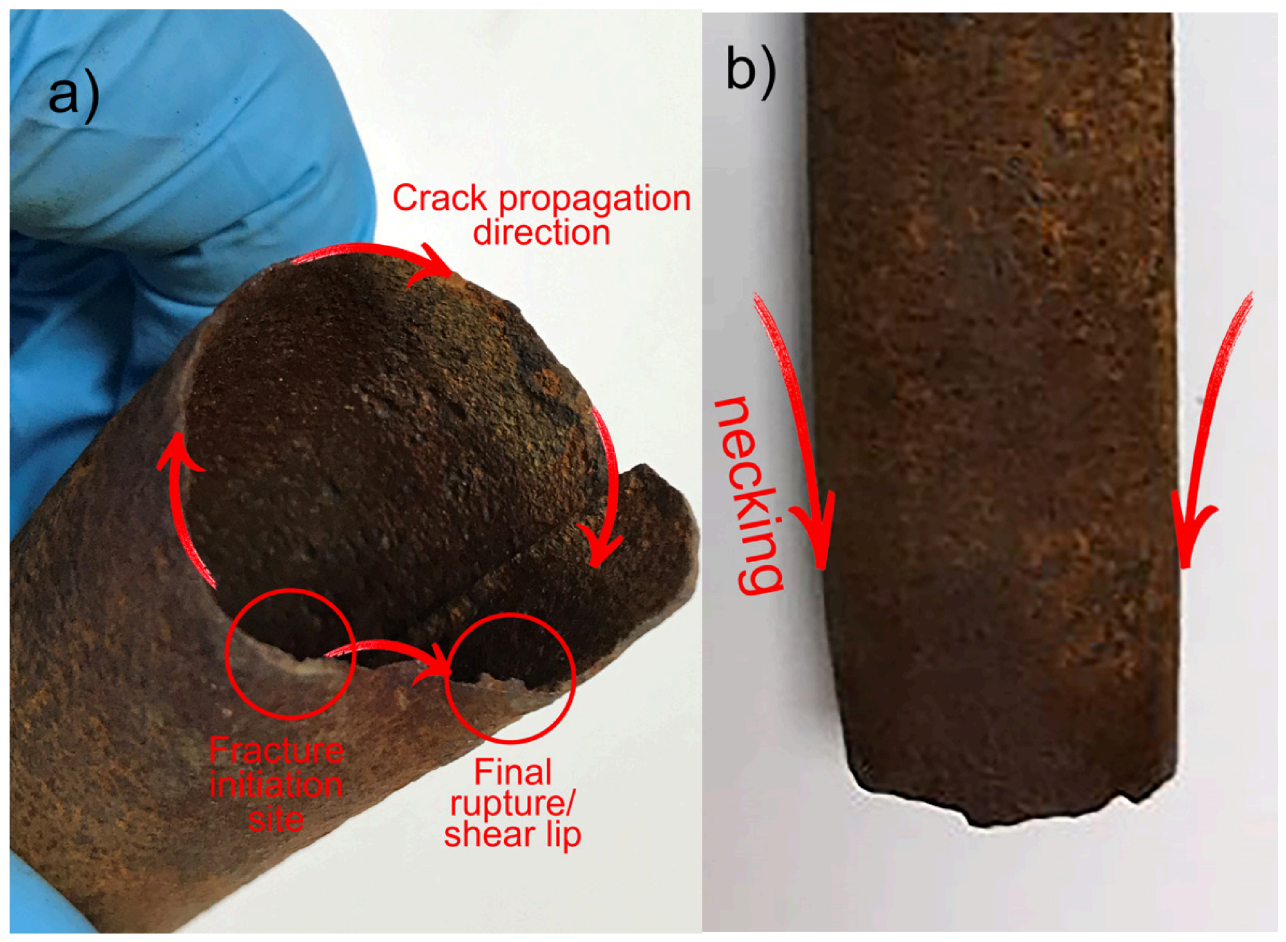
Macroscopic appearance of the failed CT-80 coiled tubing at the fracture location: (**a**) fracture wall showing the identified fracture-initiation site, crack-propagation directions, and final rupture region (shear lip); (**b**) local necking and reduction in tube diameter at the fracture site. The annotations indicate the principal macroscopic fracture features.

Figure 1a shows the fracture wall, where the fracture initiated at the locally thinnest section and propagated circumferentially in both directions before final separation in the shear-lip region. Figure 1b shows pronounced local necking and reduction in tube diameter at the fracture site, consistent with plastic deformation preceding overload failure [43,44,45].

On the inner surface of the well-exposed tubing, pitting damage was observed outside the longitudinal seam region, predominantly on the side opposite the seam. Both well-exposed samples exhibited uniform general corrosion on the outer tube wall and showed marked wall thinning compared with the new reference sample. Dimensional measurements of wall thickness and outer diameter are presented in Table 1, while the calculated corrosion wall losses are summarised in Table 2. Wall thickness and outer diameter were measured manually using a vernier caliper with a resolution of 0.01 mm on tubing sections collected at the fracture location (0 m) and approximately 100 m above the fracture. For each dimensional parameter and tubing section, five independent measurements were taken at spatially separated, accessible positions rather than repeatedly at a single point. This procedure was used to account for local dimensional variations caused by corrosion and deformation and was applied consistently to both tubing sections to enable direct comparison. The values reported in Table 1 represent the arithmetic mean of five measurements and are expressed as mean ± standard deviation (n = 5). Because the corroded surfaces were locally irregular, the reported values should be interpreted as representative section-level dimensions rather than measurements of the maximum pit depth or the absolute minimum remaining wall thickness.

A separate calibration-based estimate of measurement uncertainty was not available. Therefore, the instrument resolution and the standard deviation of the five spatially separated measurements are reported to describe the measurement resolution and local dimensional variability.

The cumulative wall-loss values presented in Table 2 were calculated from the nominal tubing dimensions and the mean measured dimensions reported in Table 1. The total wall loss was calculated as

Δ*t*_total_ = *t*_nom_ − *t*_meas_,

where *t*_nom_ is the nominal wall thickness and *t*_meas_ is the mean measured remaining wall thickness. The external wall loss was estimated from the reduction in outer diameter

Δ*t*_ext_ = (*D*_nom_ − *D*_meas_)/2,

where *D*_nom_ and *D*_meas_ are the nominal and mean measured outer diameters, respectively. The internal wall loss was then calculated by subtracting the external contribution from the total wall loss

Δ*t*_int_ = Δ*t*_total_ − Δ*t*_ext_.

For the fracture-location sample, these calculations yielded a cumulative total wall loss of 1.489 mm, consisting of approximately 1.196 mm of external wall loss and 0.292 mm of internal wall loss.

The wall loss attributed to the final 2.5 h acidizing operation was calculated by subtracting the pre-operation wall-loss values documented by the pre-operation dimensional inspection of the same tubing from the cumulative post-failure values. The pre-operation condition corresponded to a total wall loss of 0.323 mm, comprising 0.122 mm of external wall loss and 0.201 mm of internal wall loss. The resulting wall losses attributed to the final operation were therefore 1.166 mm in total, 1.074 mm externally, and 0.091 mm internally.

### 2.3. Laboratory Analyses

Three CT-80 coiled-tubing specimens were examined: an unused reference specimen of the same nominal grade and dimensions, a service-exposed specimen collected from the fracture location (0 m), and a service-exposed specimen collected approximately 100 m above the fracture. The unused reference specimen had not been exposed to the acidizing treatment and was used to establish the baseline chemical composition, microstructure, and microhardness. The two service-exposed specimens originated from the failed tubing string and had undergone the same final acidizing and retrieval operation.

The unused reference specimen was used for chemical-composition analysis, baseline metallographic examination, and baseline microhardness measurements. The two service-exposed specimens were used for dimensional measurements, metallographic examination, microhardness testing, SEM/EDS examination of the inner and outer surfaces, and FT-IR analysis of the corrosion products. Fractographic examination was performed on the fracture surface of the 0 m specimen. All laboratory analyses were performed at an ambient temperature of approximately 23 °C.

Chemical analysis of a new (unused) tubing sample was performed on an optical emission spectrometer GDS 500A LECO (LECO Corporation, St. Joseph, MI, USA). Microstructure analysis was carried out on an Olympus BX51 optical metallographic microscope (Olympus Corporation, Tokyo, Japan).

Metallographic cross-sections were prepared using conventional grinding and polishing procedures and etched for 5 s with 2% Nital. The microstructures were examined using an Olympus BX51 optical metallographic microscope with the associated image-analysis software.

SEM examination of the corroded surfaces was performed using a Quanta FEG 250 FEI field-emission scanning electron microscope (FEI Company, Hillsboro, OR, USA) equipped with an Oxford Penta FET EDS detector (Oxford Instruments NanoAnalysis, High Wycombe, UK). For the 0 m sample, two regions on the inner surface and two regions on the outer surface were examined at nominal magnifications of ×200, ×500, and ×1000. For the 100 m sample, the inner surface examination included one visibly damaged region and one comparatively less damaged region, while two regions were examined on the outer surface at the same magnifications.

Vickers microhardness measurements were performed on polished cross-sections using a Struers Duramin 2 hardness tester (Struers ApS, Ballerup, Denmark) in accordance with HRN EN ISO 6507-1:2023 [46]. An HV1 test force of 9.807 N was applied. Five indentations were made on the prepared cross-section of each tubing sample. The measurement sequence comprised two positions on one side of the longitudinal seam, one position within the longitudinal seam region, and two positions on the opposite side of the seam. The five positions were subsequently represented as the 09:00, 10:30, 12:00, 13:30, and 15:00 clock-face positions, with 12:00 corresponding to the longitudinal seam. The arithmetic mean of the five measurements was calculated for each sample.

FT-IR spectroscopic analysis was performed using a Bruker Tensor 27 spectrometer (Bruker Optics GmbH, Ettlingen, Germany) by the KBr pellet method on corrosion products removed from the inner and outer surfaces of the 0 m and 100 m samples (approximately 3 mg per pellet, dried overnight at 60 °C to remove excess moisture); each spectrum represents the mean of 64 scans at a resolution of 4 cm^−1^.

## 3. Results and Discussion

### 3.1. Chemical Composition and Microstructure

The chemical composition (Table 3) corresponds to a low-alloy steel with additions of Mo, Cr, and Nb that promote the formation of needle ferrite and martensitic–austenitic islands, consistent with typical CT-80 coiled tubing steel [47].

**Figure 2:**
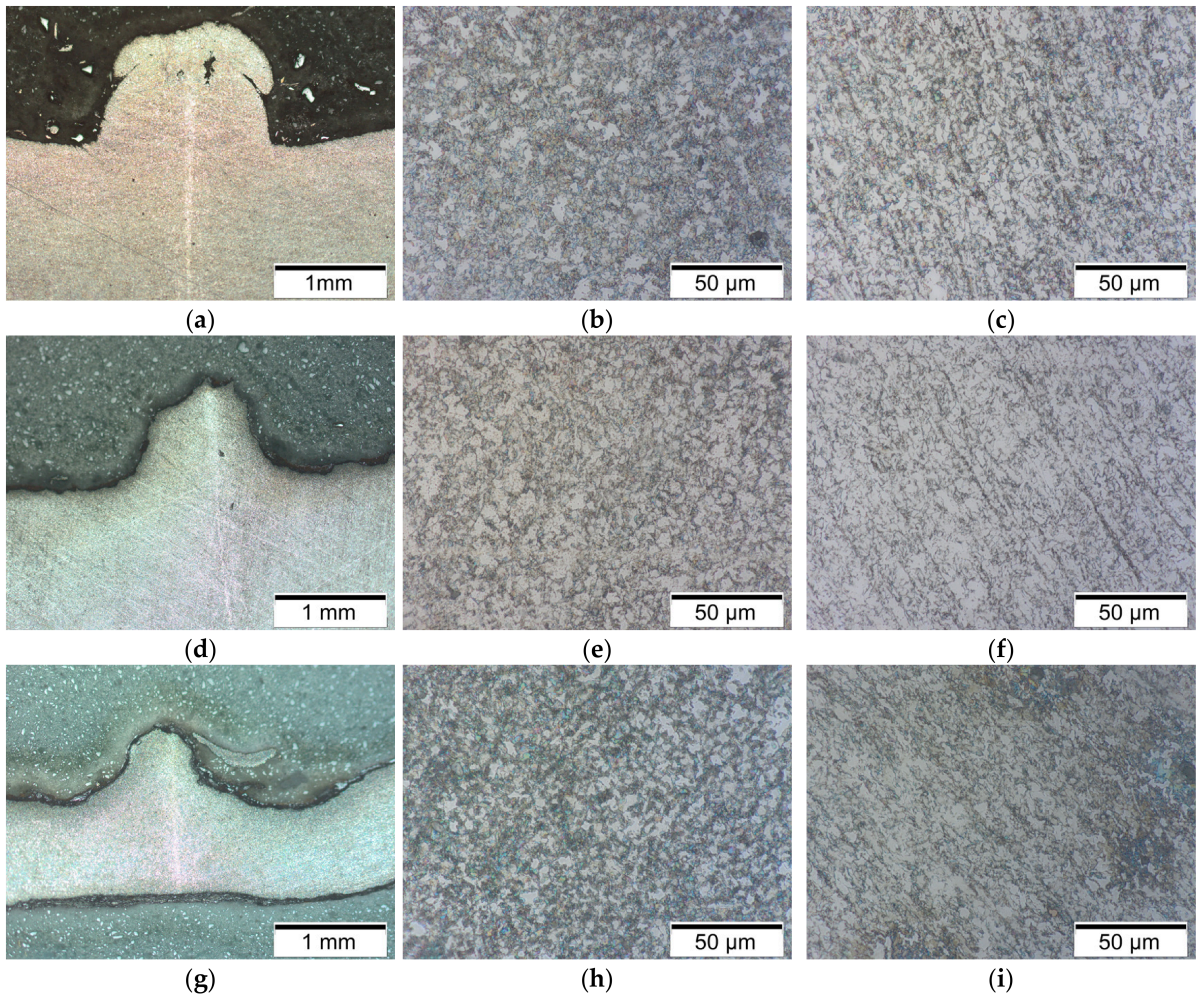
Optical micrographs of CT-80 coiled tubing etched with 2% Nital for 5 s. From left to right, the columns show the adjacent base-metal region at ×1000, the longitudinal seam region at ×1000, and the longitudinal seam cross-section at ×200. The rows show the unused reference tubing, the specimen collected 100 m above the fracture, and the specimen collected at the fracture location (0 m), respectively. (**a**) Unused reference tubing, Longitudinal seam, ×200; (**b**) Unused reference tubing, Seam region, ×1000; (**c**) Unused reference tubing, Base-metal region, ×1000; (**d**) 100 m above the fracture, Longitudinal seam, ×200; (**e**) 100 m above the fracture, Seam region, ×1000; (**f**) 100 m above the fracture, Base-metal region, ×1000; (**g**) Fracture location (0 m), Longitudinal seam, ×200; (**h**) Fracture location (0 m), Seam region, ×1000; (**i**) Fracture location (0 m), Base-metal region, ×1000.

Figure 2 presents cross-sectional and higher-magnification optical micrographs of the unused reference tubing and the two service-exposed specimens. Panels (a), (d), and (g) show the longitudinal manufacturing seam at ×200 for the unused reference specimen, the specimen collected 100 m above the fracture, and the specimen collected at the fracture location (0 m), respectively. Panels (b), (e), and (h) show the corresponding longitudinal seam regions at ×1000, while panels (c), (f), and (i) show the adjacent base-metal regions at ×1000. The base metal exhibits a fine-grained ferritic microstructure with a rolling-induced directional arrangement of ferrite–cementite bands, whereas the longitudinal seam regions exhibit a comparatively isotropic microstructure. The microstructure and texture of high-strength strip steels are strongly influenced by thermomechanical processing and subsequent cooling conditions [48,49]. No cracks or major metallurgical defects were observed in the examined longitudinal seam or adjacent base-metal regions. Therefore, the available metallographic evidence does not support a pre-existing manufacturing-seam defect as the primary cause of failure.

### 3.2. Cross-Section Microhardness

**Figure 3:**
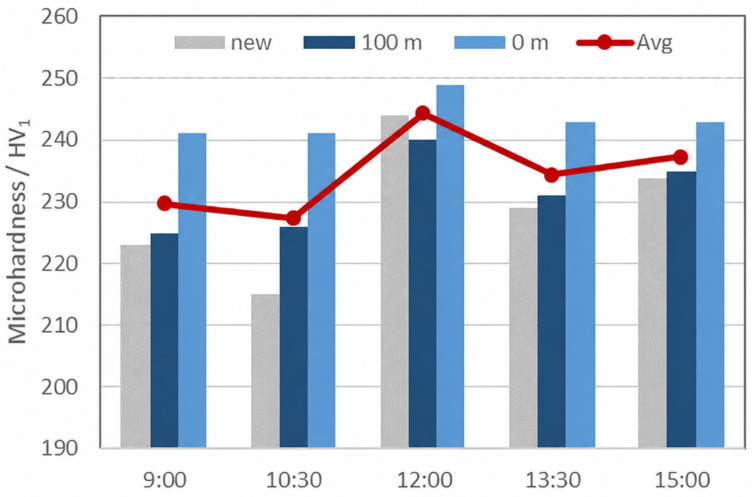
Vickers microhardness (HV1) measured at five circumferential positions in the unused reference tubing, the specimen collected 100 m above the fracture, and the specimen collected at the fracture location (0 m). The 12:00 position corresponds to the longitudinal seam.

Figure 3 presents the microhardness values graphically. The longitudinal seam region generally exhibited higher hardness than the adjacent base-metal positions, consistent with the local thermal history associated with tubing manufacture. The well-exposed specimens show higher hardness than the new sample, and the sample at the fracture location (0 m, deeper in the well) shows higher hardness than the sample at 100 m above the fracture. The red line represents the mean hardness of the three specimens at each circumferential position. The Vickers microhardness values measured at the five clock-face positions relative to the longitudinal seam are summarized in Table 4.

The depth-dependent hardness increase, from a mean value of 229 HV1 in the new tubing to 243.4 HV1 at the fracture location, may reflect several service-induced effects, including limited hydrogen uptake during acid exposure. During acid corrosion, cathodic reduction of hydrogen ions provides a source of atomic hydrogen that may enter and diffuse through the steel lattice. The presence of H_2_S in the well fluid (300 ppm detected at the surface) can inhibit the recombination of atomic hydrogen into H_2_ molecules, thereby promoting hydrogen entry into the metal [50,51,52]. McCoy [53] reported that coiled-tubing steel may become hydrogen-charged at downhole temperatures and subsequently fracture at the gooseneck during cooling and retrieval. However, microhardness is not a hydrogen-specific indicator. Repeated bending and straightening of the coiled tubing, service-induced strain hardening, and residual stresses associated with tubing manufacture, formation of the longitudinal seam, and passage over the gooseneck may also have contributed to the measured hardness increase. The relative contributions of these effects cannot be quantified from the available measurements.

The maximum measured hardness of 249 HV1, recorded in the longitudinal seam region at the fracture location, approached but did not exceed the commonly referenced 250 HV threshold associated with increased SSC susceptibility in pipeline steels [54]. Whether the same criterion should be applied directly to coiled tubing remains unclear [53]. Although the measured hardness increase indicates a change in the local mechanical response of the material, its magnitude was relatively modest and cannot be attributed exclusively to hydrogen uptake. Moreover, as discussed below, no clear fractographic evidence of a hydrogen-assisted brittle fracture mechanism was observed.

### 3.3. SEM/EDS Analysis of Corroded Surfaces

**Figure 4:**
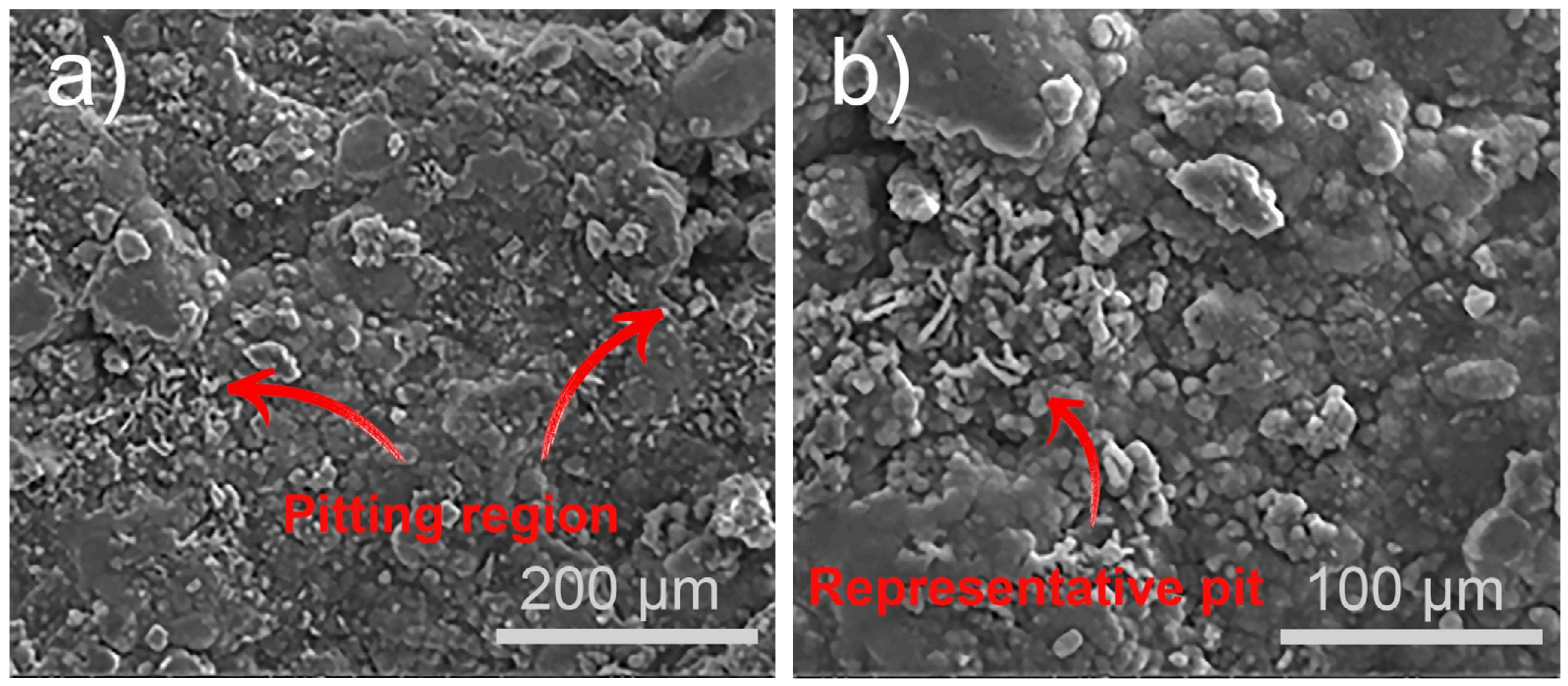
Representative SEM micrographs of pitting damage on the inner tube surface at the fracture location (0 m): (**a**) ×500, showing the broader pitting region; (**b**) ×1000, showing a representative pit and the associated rough corrosion-product morphology. The arrows indicate the analysed pitting features.

**Figure 5:**
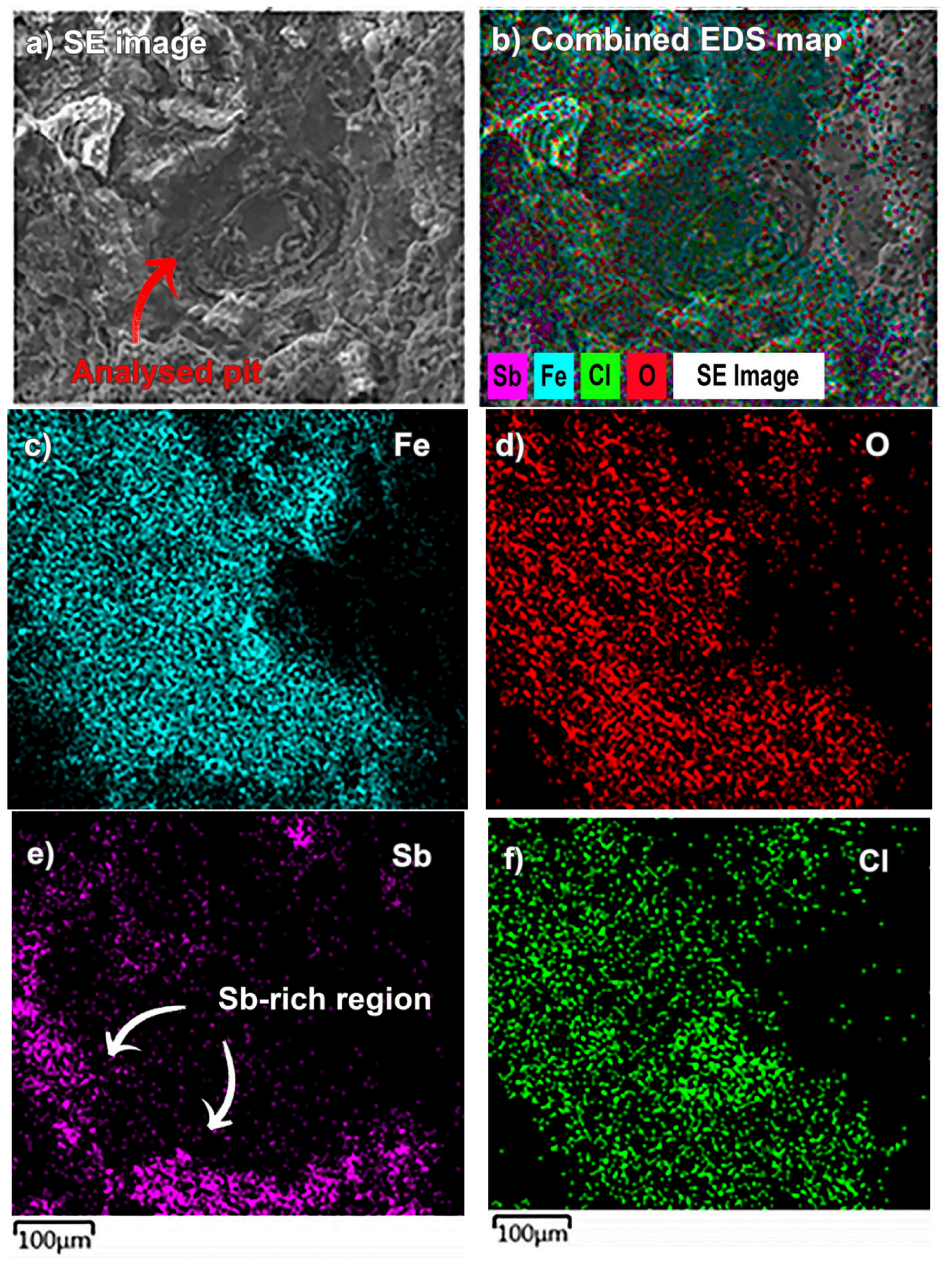
SEM/EDS analysis of a representative pitting site on the inner tube wall at the fracture location (0 m, ×500): (**a**) secondary-electron image of the analysed pit; (**b**) combined EDS map; (**c**) Fe; (**d**) O; (**e**) Sb; and (**f**) Cl. All panels correspond to the same field of view. Sb-rich regions are concentrated around the pit, whereas Fe, O, and Cl predominate within the pit region.

Scanning electron microscopy with energy-dispersive X-ray spectroscopy (SEM/EDS) was used to characterize the corroded inner and outer tube surfaces and to map the elemental distribution around pitting sites. Representative results are shown in Figure 4 and Figure 5. The presented micrographs and elemental maps were selected as representative regions from the examined areas. They illustrate local pit-associated elemental distributions and should not be interpreted as evidence of homogeneous Sb distribution across the entire inner tube surface.

Figure 5 shows the EDS mapping of a representative pitting-damage site on the inner wall. The secondary-electron image (Figure 5a) shows the analysed pit corresponding to the mapped field of view, while the combined EDS map (Figure 5b) superimposes all detected elements on the same area. The individual elemental maps show that Sb-rich regions are concentrated around the analysed pit, whereas Fe, O, and Cl predominate within the pit region.

The local EDS results presented in Table 5 confirm the presence of Sb-containing deposits on the inner surface. In the analysed regions of the 0 m inner surface, Sb contents ranged from 3.00 to 14.85 wt%, while Fe and O were the major detected elements. These results are consistent with heterogeneous Sb-containing deposits associated with Fe- and O-rich corrosion products. At measurement point SP10, the comparatively high Sb content of 14.85 wt%, together with the lower Fe content of 36.32 wt%, indicates a locally Sb-rich deposit. Because EDS analyses only a restricted surface region, these values represent the local composition of the selected measurement points and not the average composition of the entire inner surface.

The variation in elemental contents between the analysed points reflects the heterogeneous nature of the corrosion deposits. The reported EDS mass fractions should therefore be interpreted as local compositions of the selected points or fields rather than as bulk or surface-average compositions. Direct quantitative comparison between individual locations should also be made with caution because of local surface roughness, corrosion-product morphology, contamination, and elemental segregation.

On the outer surface of the 0 m sample, the EDS result at SP1 shows S (1.73 wt%) and Cr (1.28 wt%) in addition to Fe and O. This local composition is consistent with a mixed corrosion-product deposit containing Fe-, O-, S-, and Cr-bearing constituents. The dark deposit examined on the 100 m outer surface contained S (6.25 wt%), Cr (6.63 wt%), Sb (6.88 wt%), Ni (1.46 wt%), and Cu (2.91 wt%), indicating a chemically heterogeneous corrosion-product layer that may contain contributions from the inhibitor package, the tubing material, and the H_2_S-containing acidic environment.

Taken together with the reported behaviour of Sb-containing acid-inhibitor intensifiers, the local EDS observations are consistent with the following interpretation. On the inner wall, incomplete inhibitor coverage at 196 °C may have allowed localized corrosion to initiate. The concentration of Sb-rich deposits around some pits could have created local electrochemical heterogeneity between the Sb-rich regions and the exposed steel, potentially promoting anodic dissolution within the pit. However, EDS provides compositional and spatial information only; it does not directly demonstrate galvanic current flow or establish the electrochemical role of the Sb-rich deposits. The proposed Sb–Fe galvanic contribution should therefore be regarded as a mechanism supported by the observed elemental distribution and the relevant literature, rather than as a directly verified process. On the outer wall, the extensive uniform corrosion is consistent with exposure to spent, inhibitor-depleted backflow fluid under high-temperature H_2_S/CO_2_-containing conditions.

### 3.4. FT-IR Analysis of Corrosion Products

**Figure 6:**
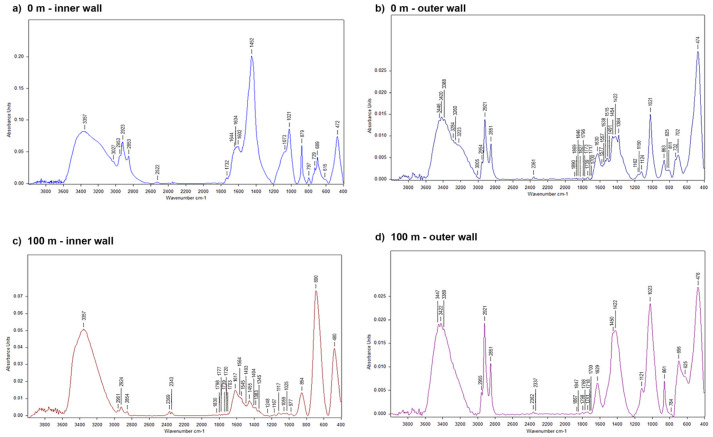
FT-IR spectra (KBr pellet method; mean of 64 scans at 4 cm^−1^ resolution; pellets dried overnight at 60 °C) of corrosion products removed from the tube surfaces: (**a**) 0 m, inner wall; (**b**) 0 m, outer wall; (**c**) 100 m, inner wall; and (**d**) 100 m, outer wall. The principal bands are tentatively assigned to O–H vibrations of hydrated oxides/oxyhydroxides, C–H vibrations of adsorbed organic species, carbonate-bearing products, and Fe–O vibrations. Bands in the 1120–1160 cm^−1^ region on the outer-wall samples are consistent with sulfate-bearing species, while stronger absorption near 690 cm^−1^ on the inner-wall samples may be associated with chloride-containing oxyhydroxides and/or Sb–O vibrations.

To complement the elemental information obtained by SEM/EDS, the corrosion products removed from the inner (pitted) and outer (uniformly corroded) surfaces of the 0 m and 100 m samples were examined by Fourier-transform infrared (FT-IR) spectroscopy. Whereas EDS identifies the elements present locally, FT-IR is sensitive to molecular bonding in oxides, oxyhydroxides, carbonates, sulfates, and adsorbed organic species and can therefore support tentative assignment of the corrosion-product phases. The four spectra are shown in Figure 6, and the principal band assignments are summarised below (all values in cm^−1^).

All four spectra share a common set of features. The broad envelope between approximately 3350 and 3450 cm^−1^ (e.g., 3357 in the inner-wall samples and the 3388–3447 group in the outer-wall samples) corresponds to O–H stretching of structural hydroxyl groups in iron oxyhydroxides together with adsorbed and hydrate water, while the weaker band near 1630 cm^−1^ (1617–1634) is the associated H–O–H bending mode [55]. The paired bands near 2920 and 2851 cm^−1^ in every spectrum are the asymmetric and symmetric C–H stretches of aliphatic CH_2_/CH_3_ groups, and together with the carbonyl absorptions in the 1700–1740 cm^−1^ region (e.g., 1732, 1739, 1717) they indicate a thin adsorbed organic residue—most plausibly remnants of the propargyl-alcohol/quaternary-ammonium/formic-acid inhibitor package and co-produced hydrocarbons. The strong low-frequency bands at 472–480 cm^−1^ (with shoulders near 615–625 cm^−1^) are Fe–O lattice vibrations of iron oxides. The narrow bands near 2340–2360 cm^−1^ arise from atmospheric/adsorbed CO_2_ and are consistent with the CO_2_-rich well gas.

A second group of bands is consistent with carbonate-containing products. The triad near 1452, 879, and 729–698 cm^−1^ in the 0 m inner-wall spectrum (Figure 6a) can be assigned, respectively, to the ν_3_ asymmetric stretch, the ν_2_ out-of-plane bend, and the ν_4_ in-plane bend of the carbonate ion (CO_3_^2−^); analogous bands appear in all spectra (e.g., 1450/1422, 861–863, and 702–732 cm^−1^). The possible formation of iron carbonate (siderite-type) is consistent with the high CO_2_ content measured in the well gas (11.29%), which may favour FeCO_3_ formation as one component of the corrosion-product layer. The intense, sharp carbonate-related features on the 0 m inner wall indicate that the inner surface, exposed to the injected fluid, accumulated a substantial carbonate-bearing scale.

The outer-wall spectra (Figure 6b,d) are distinguished by additional bands in the 1120–1160 cm^−1^ region (1124, 1150, and 1162 cm^−1^ at 0 m; 1121 cm^−1^ at 100 m), which are tentatively assigned to the ν_3_ asymmetric stretch of sulfate-bearing species. These bands are absent or much weaker on the inner walls and correlate with the sulfur detected by EDS on the outer surfaces (1.73 wt% at 0 m, point SP1; 6.25–7.75 wt% at 100 m; Table 5). Such sulfate-bearing products may arise through oxidation of sulfide species generated when the H_2_S-bearing well fluid (300 ppm H_2_S) reacts with the steel; the FT-IR signature therefore provides supporting evidence that the outer wall was exposed to the H_2_S-containing environment. The inner-wall spectra, by contrast, show stronger absorption near 690 cm^−1^ (a dominant band at 690 cm^−1^ in the 100 m inner-wall sample, Figure 6c), which may be associated with chloride-bearing iron oxyhydroxide and/or Sb–O vibrations [55,56]. This interpretation is consistent with the high local chlorine content detected inside the inner-wall pits by EDS (7.36 wt% at point SP23; Table 5) and the high local Sb content at the same site (31.0 wt% at SP25).

Taken together, the FT-IR spectra are consistent with the presence of iron oxide/oxyhydroxide, carbonate-bearing, sulfate-bearing, and chloride-associated corrosion products, together with thin adsorbed organic residues. These assignments are based on characteristic absorption bands reported in the literature and should be regarded as tentative phase assignments rather than definitive phase identifications [55,56]. Because corrosion layers may contain nanophase iron oxides and phases with overlapping or ambiguous spectroscopic signatures, reliable phase identification generally requires an integrated analytical approach using complementary structural techniques such as X-ray diffraction and Raman spectroscopy [57,58]. Within this limitation, the spectra support the SEM/EDS-based interpretation that effective inhibitor protection was reduced during service.

### 3.5. Fracture Analysis

Fracture is the most frequent type of coiled tubing failure; due to the variety of possible fracture mechanisms, fractographic analysis is essential for identifying the root cause [59].

**Figure 7:**
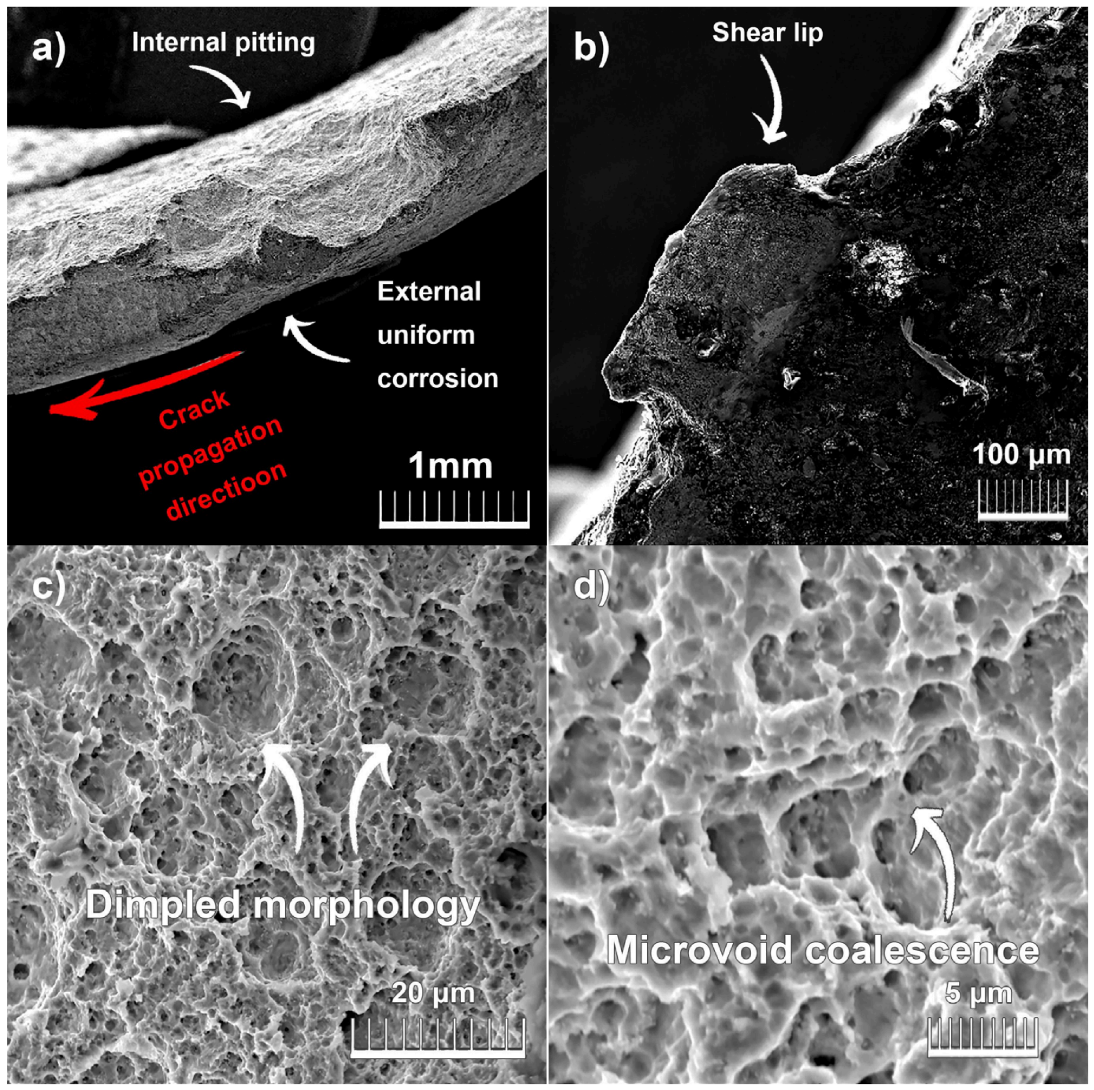
Representative SEM micrographs of the fracture morphology: (**a**) corrosion-thinned fracture wall showing internal pitting, external uniform corrosion, and the indicated crack-propagation direction; (**b**) final detachment region showing the shear lip; (**c**) dimpled fracture morphology at ×2000; and (**d**) microvoid coalescence at ×5000. The morphologies observed in panels (**c**,**d**) are characteristic of a predominantly ductile fracture mechanism.

Figure 7a shows the wall at the fracture site thinned by combined external uniform and internal pitting corrosion. Figure 7b shows the detachment site (shear lip) at the end of the fracture path. The macroscopic fracture path identified in Figure 1 is consistent with the SEM observations in Figure 7, which show a corrosion-thinned wall, a final shear-lip region, and dimpled microvoid coalescence.

In the case of hydrogen-assisted fracture, the crack path can be intergranular or transgranular, and ductile fracture in low-strength steels is characterized by dimpled microvoid coalescence (MVC). MVC can be superficially similar in appearance to hydrogen-embrittled fracture surfaces, so fractographic and metallographic results must be interpreted together and at multiple magnification levels [60,61].

Figure 7c and Figure 7d show the representative fracture-surface morphology at ×2000 and ×5000, respectively. The examined regions are dominated by dimpled microvoid coalescence, which is characteristic of a predominantly ductile fracture mechanism. No intergranular fracture, cleavage facets, or other clear brittle-fracture features were observed in the analysed regions. Nevertheless, because the hydrogen content of the steel was not directly measured, a limited contribution of hydrogen to local ductility reduction cannot be fully excluded. The available fractographic and metallographic evidence therefore supports ductile overload of a corrosion-thinned section as the primary failure mechanism, rather than a purely hydrogen-driven brittle fracture.

The fracture mechanism is therefore interpreted as progressive corrosion-assisted loss of the load-bearing cross-section, followed by mechanical overload during retrieval. External uniform corrosion and internal localized pitting reduced the remaining wall thickness. During passage through the gooseneck, the combined tensile and bending loads exceeded the capacity of the thinned section, resulting in predominantly ductile overload fracture. Hydrogen uptake may have contributed to a limited reduction in ductility, but its magnitude and specific contribution could not be determined in the absence of direct hydrogen measurements.

For future HTBH acidizing operations, this case shows that inhibitor qualification should not be based only on fresh inhibited acid exposure. The backflow stage must also be reproduced because the returning acid may be spent, inhibitor-depleted, enriched with dissolved corrosion products, and still highly corrosive. Laboratory testing should therefore include the actual bottom-hole temperature, realistic exposure time, H_2_S/CO_2_ chemistry, and a representative acid-volume-to-metal-surface-area ratio. In addition, the inner and outer tube surfaces should be evaluated separately, since the present failure demonstrates that they can experience different corrosion mechanisms during the same operation. Post-acidizing inspection should include wall-thickness measurement and pit-depth assessment before further reuse, particularly when the operation approaches 200 °C or when H_2_S is detected. From a failure-prevention perspective, the critical control parameter is not only the nominal inhibitor concentration in the injected acid, but also the remaining protection level during the backflow stage and during retrieval through the gooseneck.

## 4. Conclusions

The following conclusions are drawn from this investigation:

1. The failure developed in a severely and asymmetrically corrosion-thinned section of the CT-80 tubing. The outer wall was affected predominantly by uniform corrosion, whereas the inner wall exhibited localized pitting. During the final acidizing operation, the external wall loss was approximately 11.8 times greater than the internal wall loss, indicating markedly different exposure conditions on the two surfaces.
2. The combined dimensional, SEM/EDS, and FT-IR results are consistent with reduced inhibitor protection during spent-acid backflow and incomplete protection of the inner surface at 196 °C. Sb-rich deposits around individual pits may have contributed to local electrochemical acceleration of corrosion; however, this mechanism remains interpretative because galvanic coupling was not measured directly.
3. Necking, diameter reduction, and dimpled microvoid coalescence indicate that the primary failure mode was ductile overload during retrieval through the gooseneck after corrosion had substantially reduced the load-bearing cross-section. Hydrogen may have contributed to the measured hardness increase or to a limited reduction in ductility, but a hydrogen-assisted brittle fracture mechanism was not demonstrated.
4. For HTBH acidizing operations, inhibitor qualification should reproduce not only exposure to fresh inhibited acid but also the spent-acid backflow stage under realistic temperature, exposure time, H_2_S/CO_2_ chemistry, and acid-volume-to-metal-surface-area conditions. Inspection protocols should assess the inner and outer tubing surfaces separately and include both remaining wall-thickness and pit-depth measurements before further tubing reuse.

## Figures and Tables

**Table 1:** Mean wall thickness and outer diameter measured manually at five spatially separated points on tubing sections collected at the fracture location and 100 m above the fracture. Values are reported as mean ± standard deviation (n = 5).

Wall Thickness 0 m from Fracture/mm	Wall Thickness 100 m from Fracture/mm	Outer Diameter 0 m from Fracture/mm	Outer Diameter 100 m from Fracture/mm
1.101 ± 0.085	2.018 ± 0.048	29.357 ± 0.346	30.940 ± 0.337

**Table 2:** Cumulative corrosion wall loss and wall loss attributed to the final 2.5 h acidizing operation, calculated from dimensional measurements (nominal wall thickness: 2.590 mm; nominal outer diameter: 31.750 mm).

Parameter	Unit	Cumulative (All Operations)	Final Operation (2.5 h Exposure)
Total wall loss	mm	1.489	1.166
External wall loss	mm	1.196	1.074
Internal wall loss	mm	0.292	0.091
Ratio (ext/int)	—	~4.1×	~11.8×

**Table 3:** Chemical composition of coiled tubing measured by GDS optical emission spectrometry.

**C (%)**	**Mn (%)**	**Si (%)**	**P (%)**	**S (%)**	**Mo (%)**	**Ni (%)**	**Cr (%)**	**Cu (%)**
0.163	0.743	0.371	0.010	0.0021	0.124	0.146	0.574	0.277
**W (%)**	**Al (%)**	**Ti (%)**	**Co (%)**	**Nb (%)**	**Sn (%)**	**As (%)**	**Zr (%)**	
0.0038	0.0375	0.014	0.0025	0.0187	0.0051	0.0063	0.0019	

**Table 4:** Vickers microhardness (HV1) of coiled tubing samples at positions relative to the longitudinal seam. Positions are expressed as clock-face hours (12:00 = seam).

Position (Clock-Face)	New Tubing (HV1)	100 m from Fracture (HV1)	0 m from Fracture (HV1)	Increase (New → 0 m)
09:00	223	225	241	+8.1%
10:30	215	226	241	+12.1%
12:00 (seam)	244	240	249	+2.1%
13:30	229	231	243	+6.1%
15:00	234	235	243	+3.8%
Mean value	229	231.4	243.4	+6.3%

**Table 5:** Representative EDS results (mass%) at selected measurement points on inner and outer tube surfaces at 0 m and 100 m positions.

Position/Location	Point	C (%)	O (%)	Cl (%)	S (%)	Cr (%)	Fe (%)	Cu (%)	Sb (%)
0 m inner	SP9	6.28	39.14	0.41	—	—	50.23	—	3.00
0 m inner	SP10	8.95	39.57	0.31	—	—	36.32	—	14.85
0 m inner	SP11	—	41.78	0.61	—	—	50.72	—	5.74
0 m outer	SP1	5.64	43.47	—	1.73	1.28	46.39	0.55	—
0 m outer	SP3	6.68	35.59	—	—	—	57.73	—	—
0 m outer	SP4	8.17	35.56	—	—	1.28	52.24	—	2.75
100 m inner (pit)	SP23	—	17.29	7.36	—	—	75.35	—	—
100 m inner (pit)	SP25	10.69	26.36	1.95	—	—	29.98	—	31.02
100 m outer (dark)	—	12.67	21.26	—	6.25	6.63	40.37	2.91	6.88

## Data Availability

The original contributions presented in this study are included in the article. Further inquiries can be directed to the corresponding author.
